# Predicting individual contrast sensitivity functions from acuity and letter contrast sensitivity measurements

**DOI:** 10.1167/16.15.15

**Published:** 2016-12-22

**Authors:** Steven M. Thurman, Pinakin Gunvant Davey, Kaydee Lynn McCray, Violeta Paronian, Aaron R. Seitz

**Affiliations:** steven.matthew.thurman@gmail.com; pdavey@westernu.edu; kmccray@westernu.edu; vparonian@westernu.edu; aseitz@ucr.edu; U.S. Army Research Laboratory, Human Research and Engineering Directorate, Los Angeles, CA, USA; College of Optometry, Western University of Health Sciences, Pomona, CA, USA; College of Optometry, Western University of Health Sciences, Pomona, CA, USA; College of Optometry, Western University of Health Sciences, Pomona, CA, USA; Department of Psychology, University of California, Riverside, CA, USA

**Keywords:** *contrast sensitivity*, *visual acuity*, *contrast sensitivity function*, *modeling*, *individual differences*

## Abstract

Contrast sensitivity (CS) is widely used as a measure of visual function in both basic research and clinical evaluation. There is conflicting evidence on the extent to which measuring the full contrast sensitivity function (CSF) offers more functionally relevant information than a single measurement from an optotype CS test, such as the Pelli–Robson chart. Here we examine the relationship between functional CSF parameters and other measures of visual function, and establish a framework for predicting individual CSFs with effectively a zero-parameter model that shifts a standard-shaped template CSF horizontally and vertically according to independent measurements of high contrast acuity and letter CS, respectively. This method was evaluated for three different CSF tests: a chart test (CSV-1000), a computerized sine-wave test (M&S Sine Test), and a recently developed adaptive test (quick CSF). Subjects were 43 individuals with healthy vision or impairment too mild to be considered low vision (acuity range of −0.3 to 0.34 logMAR). While each test demands a slightly different normative template, results show that individual subject CSFs can be predicted with roughly the same precision as test–retest repeatability, confirming that individuals predominantly differ in terms of peak CS and peak spatial frequency. In fact, these parameters were sufficiently related to empirical measurements of acuity and letter CS to permit accurate estimation of the entire CSF of any individual with a deterministic model (zero free parameters). These results demonstrate that in many cases, measuring the full CSF may provide little additional information beyond letter acuity and contrast sensitivity.

## Introduction

The spatial contrast sensitivity function (CSF) represents a useful summary of functional vision by measuring the amount of contrast needed to detect or discriminate patterns across a range of spatial scales (Campbell & Robson, 1968). The clinical importance of contrast sensitivity (CS) is supported by research showing that many conditions, including amblyopia (Bradley & Freeman, 1981; Hess & Howell, 1977), glaucoma (Hot, Dul, & Swanson, 2008; Ross, Bron, & Clarke, 1984), macular degeneration (Kleiner, Enger, Alexander, & Fine, 1988), diabetic retinopathy (Della Sala, Bertoni, Somazzi, Stubbe, & Wilkins, 1985; Sokol et al., 1985), and cataracts (Chylack et al., 1993; Drews-Bankiewicz, Caruso, Datiles, & Kaiser-Kupfer, 1992), show measurable losses in CS, even in cases where acuity may be in the normal range (Bodis-Wollner, 1972; Jindra & Zemon, 1989; Regan & Neima, 1984; Woods & Wood, 1995). Impairment of CS is also associated with functional disabilities (Rubin & Legge, 1989; Turano, Rubin, & Quigley, 1999), and is often more predictive of performance impairment than are standard acuity measurements (Brabyn, Schneck, Haegerstrom-Portnoy, & Lott, 2001; Ginsburg, 2003; Ginsburg, Evans, Sekule, & Harp, 1982).

Although numerous methodologies have been developed over the decades to measure threshold CS, the standard approach presents single targets (e.g., sine-wave gratings or letter optotypes) and measures the smallest amount of luminance contrast needed to detect or discriminate the target using various tasks that include target detection, letter identification, and orientation discrimination. Optotype-based tests, prominent examples being the Pelli–Robson chart (Pelli, Robson, & Wilkins, 1988) and the Mars chart (Dougherty, Flom, & Bullimore, 2005), are more commonly used in the clinic due to time efficiency and ease of use. While these tests may be sensitive enough to detect generalized deficits in CS (Ismail & Whitaker, 1998; Stavrou & Wood, 2003), they generally lack power to resolve frequency-specific deficits due to the broadband nature of letter stimuli (Ginsburg, 2003; Legge, Pelli, Rubin, & Schleske, 1985). In comparison, full CSF tests use several band-limited targets, such as sine-wave gratings or Gabors, to measure CS across a broad range of spatial frequencies (Arden & Jacobson, 1978; Campbell & Robson, 1968; Ginsburg, 1984; Owsley, 2003), and may be better equipped to reveal potentially relevant changes in the shape of the CSF (Regan, Silver, & Murray, 1977).

A key question of practical importance presented in the literature on visual CS regards the extent to which unique and useful information might be gained from lengthy procedures that estimate the full range of the CSF, in comparison to time-efficient methods that produce a single CS estimate with letter optotypes (Pelli et al., 1988; Woods & Wood, 1995). This issue is often posed in terms of whether frequency-specific information can reveal subtle and functionally relevant differences between normal- and low-vision groups. While there is evidence in support of measuring the full CSF to distinguish certain low-vision groups (Bour & Apkarian, 1996; Hess & Howell, 1977; Kupersmith, Seiple, Nelson, & Carr, 1984), there is also evidence to show redundancy in the information provided by these techniques (Elliott & Whitaker, 1992; Woods & Wood, 1995). In fact, Chung and Legge (2016) recently proposed a provocative hypothesis regarding this issue, suggesting that the CSF curve of an individual might be accurately estimated by simply shifting a standard “template” CSF horizontally and vertically along the log axes according to independent measures of high-contrast visual acuity and letter CS, respectively. If the full range of an individual CSF can be accurately recovered from these two auxiliary measures, then this would suggest redundancy with more common, and efficient, clinical measures of visual function and, as a practical matter, may obviate the apparent need in many cases to measure the full CSF.

The present study examined this issue within a heterogeneous cohort of observers with substantial variance in age, visual function, and history of ophthalmologic disease. We conducted three distinctive tests designed to estimate the CSF: the CSV-1000 chart-based test (Vector Vision Co., Greenville, OH), a computerized sine-grating test (M&S Technologies, Dallas, TX), and quick CSF (Adaptive Sensory Technology, Inc., Boston, MA), which offers a novel adaptive approach to estimate functional parameters of the underlying CSF using a 10-alternative forced-choice task on narrowband filtered Sloan letters (Hou, Lesmes, Bex, Dorr, & Lu, 2015). In addition, broadband (unfiltered) letter CS measurements were obtained from the Pelli–Toronto test (M&S Technologies), a computerized version of the Pelli–Robson chart test, and acuity measurements were acquired with ETDRS charts at near (40 cm) and far (3 m) distances. Log CS (logCS) measurements from each CSF test were fitted with a functional form of the CSF (truncated log parabola) with four interpretable parameters corresponding to peak CS, peak spatial frequency (SF), bandwidth, and truncation to define the asymmetrical plateau at low frequencies (Carney et al., 2000; Lesmes, Lu, Baek, & Albright, 2010; Watson & Ahumada, 2005). All tests were repeated in the same or following day to assess test–retest repeatability using the Bland–Altman method (Bland & Altman, 1999) as an indicator of test reliability, and to serve as a basis for comparison to errors in model-based predictions.

We developed a predictive modeling framework that measures a template CSF from group-averaged data and uses two simple linear models to capture intrinsic relationships between letter CS and peak CSF sensitivity, and between acuity and peak SF, across the group of participants. In the overall model, a total of six free parameters are fitted to group-level data. Evaluating this model on an individual-subject basis by plugging in empirical measures of letter CS and acuity thus delivers an individualized prediction of four functional parameters and, by evaluation, frequency-specific logCS data to compare directly with raw measured data. In this way, the full range of the CSF may be predicted on an individual basis with effectively zero free parameters, because it is determined explicitly from just two common clinical measurements of visual function. Results show that while each CSF test demands a unique standard template to fit group data, likely due to methodological differences (Elliott & Whitaker, 1992; Moseley & Hill, 1994; Woods & Wood, 1995), a model that shifts the template CSF horizontally and vertically according to acuity and letter CS is capable of predicting the full range of CSF data for most individuals with roughly the same precision as test–retest reliability for a given type of CSF test.

## Methods and procedures

### Subjects

Forty-three subjects (20 women, 23 men) participated in the study and were recruited at the Eye Care Institute at the Western University of Health Sciences College of Optometry by use of advertisement with flyers and posters in the local campus community. Recruiting participants at the clinic offered the benefit of obtaining a relatively diverse sample of participants in terms of age (22 to 86 years), acuity (−0.3 to 0.34 logMAR), and history of ophthalmologic disease. While the study obtained a broad sample of subjects visiting the clinic for routine examination, it was not explicitly designed with the power to compare individuals with normal and low, due to the convenience sampling. Incidentally, 29 of the 43 subjects (mean age = 36.03 years, *SD* = 10.55) were designated as having healthy vision with no history of ophthalmic disease, and 14 of the 43 (mean age = 57.71 years, *SD* = 16.64) had prior history of some type of ophthalmic disease (seven cataracts, two glaucoma, one ocular hypertension, three age-related macular degeneration, and one amblyopia). Despite the prior history and later age of some individuals, this group collectively had a relatively mild degree of functional impairment, evidenced by the fact that all subjects had better than 20/40 vision except a single individual with 20/50 acuity. All subjects gave written informed consent in accordance with the study procedures approved by the institutional review board at the Western University of Health Sciences, and all research was done in adherence to the tenets of Declaration of Helsinki. All examinations were performed binocularly and were repeated on the same or following day, with the exception of two participants who returned within 1 week of the first examination. The order in which the examinations were completed was determined pseudorandomly for each subject with a random number generator.

### Vision examinations

#### CSF tests

The Sine CSF is a computerized test presented on a calibrated monitor and computer system, the Smart System II, produced by M&S Technologies. The test presented sine-wave targets of varying orientation (0°, 45°, 90°, and 135°) for up to 5 s at a distance of 9 ft, and subjects verbally reported the perceived orientation. Using a staircase procedure, the test measured contrast thresholds for five levels of spatial frequency: 1.5, 3, 6, 12, and 18 c/°, presented in that order. Michelson contrast ranged from 100% to 0.5%. We used the standard settings associated with the built-in Sine CSF test without modification.

The CSV-1000 is a printed chart-based test produced by VectorVision. The test presented sine-wave targets of varying spatial frequency (3, 6, 12, and 18 c/°) at a distance of 8 ft, with subjects making a forced choice between two targets presented on different rows (one with sinusoidal modulation and the other that a solid gray). Contrast was adjusted using the method of limits, with eight discrete levels of contrast per SF, and the lowest contrast available on the chart was 0.5%. We used the table listed on the company's website (http://www.vectorvision.com/csv1000-norms/) to translate scores, which range from 1 to 8, to logCS units for curve fitting and analysis. We used the standard recommended protocol associated with the commercial CSV-1000 test (without glare).

Quick CSF (qCSF) is a relatively new computerized test designed to measure a specific functional form of the CSF, the truncated log parabola, using Bayesian adaptive methods (Dorr, Lesmes, Lu, & Bex, 2013; Hou et al., 2010; Lesmes et al., 2010). The test involved 50 trials with narrow-band Sloan letters filtered in the SF domain with a raised cosine function (Chung, Legge, & Tjan, 2002) and full-bandwidth half-height value of 1 octave. Filtered letter stimuli were resized to generate 16 evenly log-spaced center SFs ranging from 1.33 to 32 c/°. Hence, the size of letter stimuli was scaled according to SF (e.g., higher SFs appear as smaller targets). Subjects performed a 10-alternative forced-choice letter-identification task at a distance of 14 ft. The SF and contrast of targets varied adaptively from trial to trial to sample the entire joint space of SF and contrast optimally according to an information criterion, and hence to provide efficient estimation of four functional parameters (Hou et al., 2015).

#### Letter CS test

The Pelli–Toronto is a computerized test on the M&S Smart System II that is designed to replicate the Pelli–Robson chart, which is classically performed on a print-based medium (Pelli et al., 1988). Recent work has found good agreement between measurements obtained with the computerized Pelli–Toronto test and the Pelli–Robson chart (Chandrakumar, Colpa, Reginald, Goltz, & Wong, 2013). Advancements of the computerized system include the ability to display a single letter at a time, to randomize the order of letters for each examination, and to change the angle of resolution (i.e., acuity level) of the letters at will. The test was performed at a distance of 9 ft, and the task was to identify unfiltered Sloan letters (size: 20/80 angle of resolution) while an adaptive staircase procedure adjusted the level of Weber contrast until threshold was estimated. Because letter size is variable for this test, we chose a letter size that exceeded the minimum angle of resolution for all individuals in the study (all subjects greater than 20/50 acuity), so that acuity would not likely be a limiting factor for CS performance. For reference, letter size for the Pelli–Toronto test in our study was 20/80, whereas letter size for the standard Pelli–Robson chart is 20/60.

#### Visual-acuity tests

High-contrast visual acuity was measured at near (40 cm) and far (3 m) distances with printed ETDRS charts using standard procedures. LogMAR was scored letter by letter (Arditi & Cagenello, 1993).

## Analysis

### Modeling the CSF

**Figure 1:**
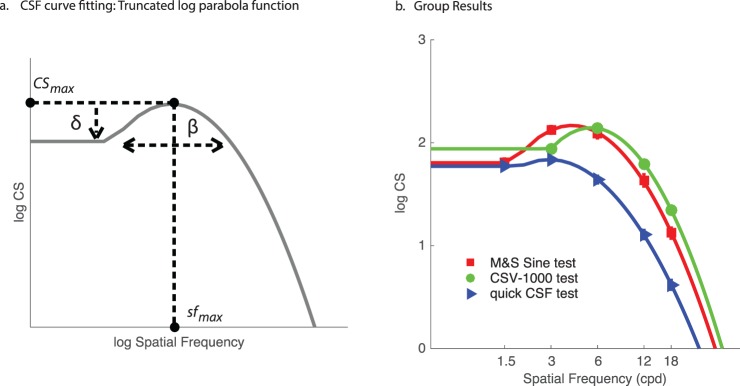
(a) Schematic illustration of the four parameters defining the truncated log-parabola function, and (b) group mean logCS data with best-fitting model curves. Error bars represent standard error of the mean.

Data were analyzed using MATLAB (Mathworks, Natick, MA). The outcome variable for each test was contrast sensitivity (1/contrast threshold) logarithmically transformed (base 10) to logCS units. For each of the three CSF tests, logCS measurements were fitted with a parametric form of the CSF (Figure 1a), the truncated log-parabola function (Carney et al., 2000; Lesmes et al., 2010). We used this function because it has been demonstrated to produce suitable fits to CSF data of individual subjects (Hou et al., 2010; Watson & Ahumada, 2005), and because the qCSF test was designed specifically to estimate parameters for this particular function (Lesmes et al., 2010). According to this model, log (decimal) sensitivity *S*(*f*) is defined by the following equations:

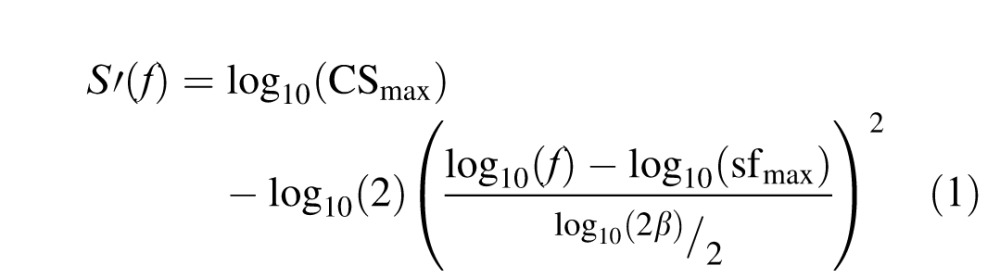


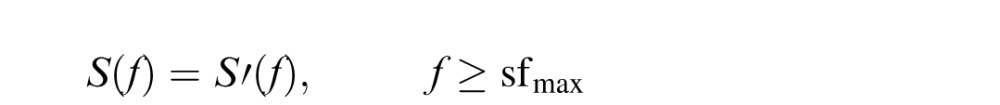


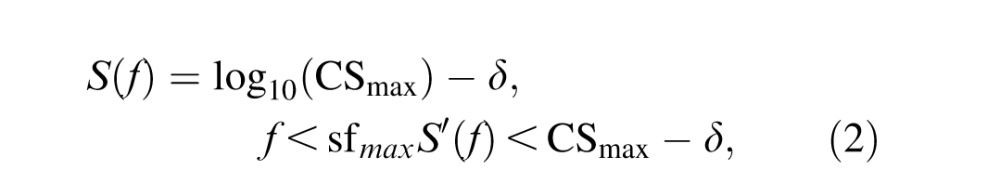
where *S*′(*f*) defines the standard three-parameter log-parabola model in Equation 1 and the rules for low-frequency truncation are specified in Equation 2.

Benefits of model fitting include the ability to summarize the entire CSF with interpretable parameters including the peak CS (CS_max_; units: CS); peak SF (sf_max_; units: c/°); bandwidth (*β*; units: c/°), describing the width of the parabola; and truncation (*δ*; units: logCS), describing the plateau of the function at the lowest SFs (Figure 1a). Curve fitting was performed in MATLAB using a built-in nonlinear least-squares regression algorithm (nlinfit.m). Parameterizing the CSF according to this function also permits estimation of the area under the log CSF (AULCSF), a summary statistic that quantifies the entire range of contrast visibility (Applegate, Hilmantel, & Howland, 1997), as well as the high-SF cutoff value (the SF where threshold is 100% contrast and therefore logCS = 0). To evaluate the quality of log-parabola model fits, we compute the root-mean-squared error (RMSE) between recorded data and model estimates evaluated at the same SFs (see Equation 8).

### Test–retest repeatability

Test–retest repeatability was evaluated using the Bland–Altman method (Bland & Altman, 1999). We computed the coefficient of repeatability (COR) as 1.96 times the standard deviation of test–retest differences (test 2 − test 1), representing the 95% level of agreement. The magnitude of COR indicates test reliability, where lesser values indicate less variability of repeated measurements and therefore better reliability. The mean difference between test and retest represents the bias.

### Relationships to letter CS and acuity

To assess the relationship between CSF model parameters and auxiliary visual measures (high-contrast acuity and letter CS), we computed the Pearson correlation coefficient to indicate the strength and directionality of the linear relationships. Prior work has shown a negative linear relationship between high-contrast acuity and peak SF of the CSF (Chung & Legge, 2016), which we sought to replicate in our sample of subjects with comparatively better baseline visual function. The negative relationship reflects the fact that subjects with better acuity will have lower values on the logMAR scale but higher values of peak SF. We also expected a positive linear relationship between letter CS and peak CS based on prior work (Pelli et al., 1988; Pelli, Rubin, & Legge, 1986), although not all studies have found this relationship to be as significant (Rohaly & Owsley, 1993). Our analysis focused on these specific relationships, but for completeness we later report the full set of correlations among all parameters and these two measurement types (Table 4).

**Table 4:**
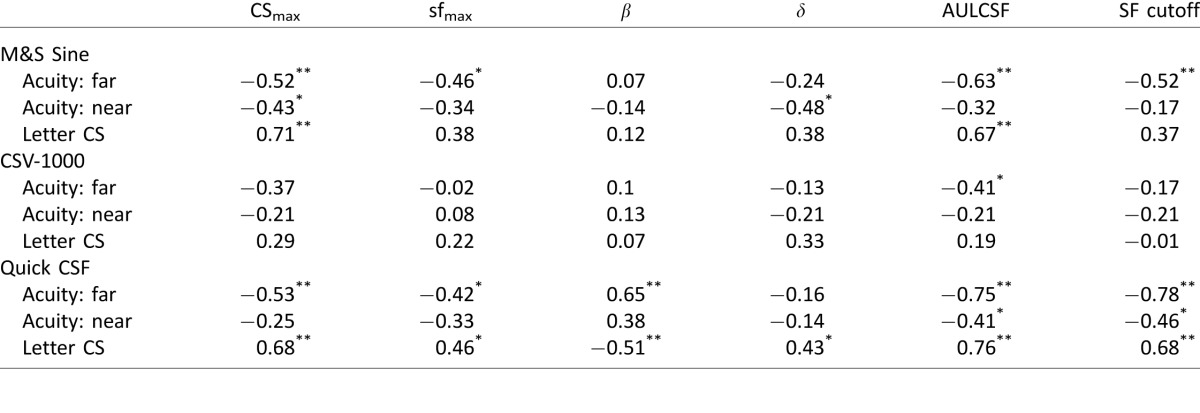
Pearson correlation coefficients between functional parameters of the truncated log-parabola model fits and auxiliary measures of visual function (far and near acuity, letter CS). *Notes*: *p < 0.05; **p < 0.01.

## Results

### Group descriptive statistics

The group means and standard deviations of logCS values for each CSF test are reported in Table 1. LogCS values were similar from test to retest for each test type, highlighting the general reproducibility of results on repeated measurements. In comparing across different CSF tests, the Sine and qCSF tests provided comparable measurements for logCS at the lowest SF (1.5 c/°). However, test results increasingly diverged for measurements at higher SFs. For the highest SF (18.0 c/°), the Sine test produced a mean measurement of 1.12 logCS, which was twice as high as the measurement from the qCSF test (0.56 logCS); this may be explained by the fact that the qCSF scaled the size of the stimuli by the SF, and hence stimuli were significantly smaller than in the other tests at high SFs. For instance, the use of smaller high-frequency stimuli presented by the qCSF could result in increased fixation errors and position uncertainty, especially near the limit of contrast visibility, which could help account for the relative reduction in logCS. The CSV-1000 produced the highest measurement (1.35 logCS at 18 c/°). In examining Table 1, there are substantial differences in the raw levels of measurement produced by the tests. The pattern of differences is not straightforward to characterize and may be better understood by fitting the raw data with the functional model of the CSF and comparing differences in functional parameters, as reported in the following section.

**Table 1:**
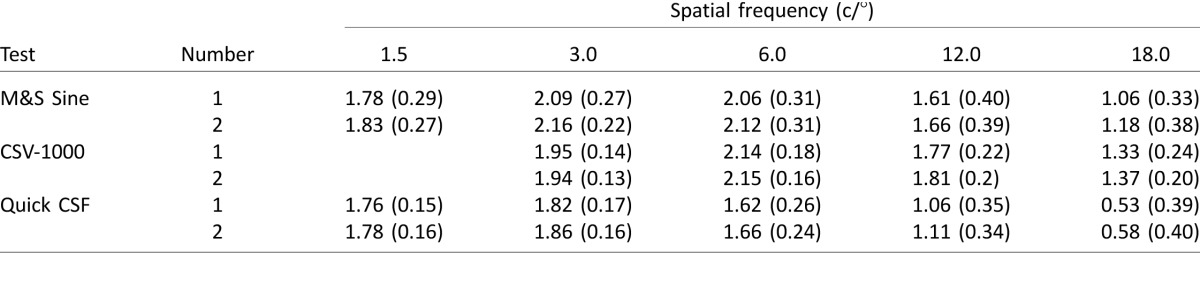
Mean (*SD*) logCS for each type of CSF test. *Notes*: Test Number 2 represents retest.

Mean logCS values for the Pelli–Toronto letter test were similar from test (*M* = 1.57, *SD* = 0.25) to retest (*M* = 1.59, *SD* = 0.25). In terms of high-contrast visual acuity, logMAR values for distance acuity ranged from −0.3 (20/10) to 0.34 (20/45), and near acuity ranged from −0.2 (20/12.5) to 0.3 (20/40). The median for both near and far acuity was −0.06 logMAR—slightly better than 20/20 vision—indicating a sample population with generally little to no deficit in high-contrast visual acuity.

### Test–retest repeatability

The Bland–Altman COR and bias are reported for each test measurement in Table 2. Among the CSF tests, the qCSF showed the best overall repeatability, with COR values ranging from 0.23 to 0.26 logCS. The CSV-1000 produced repeatable measurements with COR values ranging from 0.29 to 0.42 logCS, and the Sine test demonstrated relatively poorer repeatability, with COR values ranging from 0.41 to 0.63 logCS. The bias from test to retest was low for all test measurements, ranging from 0.01 to 0.12 logCS. Bias values tended toward the positive range, representing a minor generalized increase in logCS for retest measurements in comparison to the first test. This small improvement likely represents an effect of practice or task-specific learning (Lu, Hua, Huang, Zhou, & Dosher, 2011).

**Table 2:**
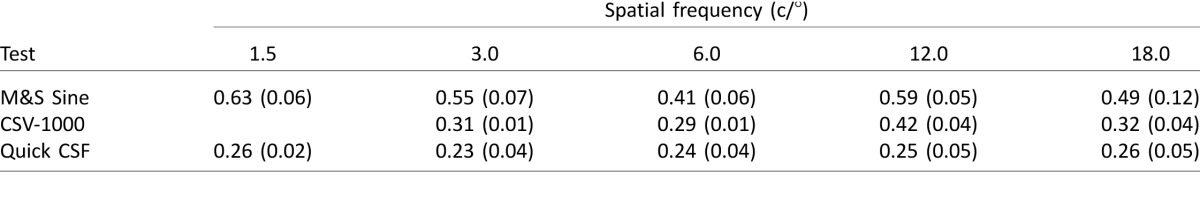
Bland–Altman coefficient of repeatability (bias) for test–retest measurements.

Like the CSF tests, the Pelli–Toronto letter CS test showed moderate repeatability (COR = 0.39) with very low bias (0.01). Generally, these levels of repeatability are comparatively similar to results of prior studies on the repeatability of other CS tests, which commonly range from about 0.15 to 0.5 logCS units (Buhren, Terzi, Bach, Wesemann, & Kohnen, 2006; Kelly, Pang, & Klemencic, 2012; Kollbaum, Jansen, Kollbaum, & Bullimore, 2014; Pomerance & Evans, 1994). Of note, the qCSF test stands out in terms of its precision and repeatability across the full spectrum of SFs, which may be attributed to methodological differences in which CS estimates are constrained by estimation of the functional form of the CSF. A result of parametric function fitting is to effectively smooth across the data (because of the nonindependence of CS estimates across SFs), a likely contributor to the increased consistency of test–retest measurements.

Due to the suitable levels of agreement between test and retest, we average these measurements for all subsequent analyses reported in this article. We will return to test–retest reliability measures in the final section as a means of evaluating the performance of the predictive model, because the precision of repeated measurements for a given test offers an upper bound on how well one could expect to be able to quantitatively predict raw measurements derived from that test.

### CSF model fits

Next we fitted raw logCS data with the truncated log-parabola function. Fitting this model resulted in an estimate for four functional parameters that specify the overall shape of the CSF (Figure 1a). The mean group results for parameter estimates are reported in Table 3. The best-fitting curves reveal the presence of significant differences in comparing results across the three tests (Figure 1b), demonstrated by the systematic shifts in functional parameters specified by the model, particularly in terms of peak CS, peak SF, and truncation.

**Table 3:**
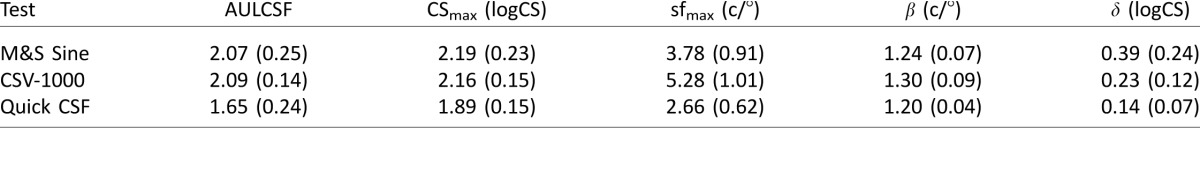
Mean (*SD*) parameter estimates for each type of CSF test.

Analyzing these differences quantitatively with a one-way analysis of variance revealed a significant effect for the peak-sensitivity parameter (CS_max_), *F*(2, 126) = 39.4, *p* < 0.001, which was due primarily to much lower values for the qCSF in comparison to the other two tests. There was a significant effect for peak SF (sf_max_), *F*(2, 126) = 127.8, *p* < 0.001, due to significantly higher values for the CSV-1000 test. The effect for bandwidth (*β*) was statistically significant, *F*(2, 126) = 29.2, *p* < 0.001, and the effect for low-SF truncation (*δ*) was also significant, *F*(2, 126) = 46.2, *p* < 0.001, with the Sine test showing significantly higher values and more variance than the other two tests. The AULCSF was also significantly different among the test types, *F*(2, 126) = 61.6, *p* < 0.001. In particular, the qCSF showed much lower AULCSF measurements in comparison to the other two tests. These analyses highlight the fact that the shape of the CSF differs systematically as a function of test type (Moseley & Hill, 1994; Woods & Wood, 1995), and helps to couch these differences in terms of interpretable functional parameters.

### Significance of functional parameters for individual model fitting

In addition to comparing functional CSF parameters at the group level among tests, it is important to benchmark in general how well the truncated log-parabola model fitted raw data of individual subjects (Supplementary Figure S1). A related issue that is pertinent to the current study concerns how important each individual parameter may be for characterizing individual differences in CS. To address this question, we performed a set of analyses in which we would fix a particular parameter of the truncated log-parabola model to the group mean value and proceed to fit the remaining three free parameters to individual data, resulting in an RMSE estimate for the partial models (i.e., missing the fixed parameter) expressed in comparable units. Parameters that are most important for capturing individual differences will result in increased RMSE relative to the full four-parameter model when fixed to the group mean. By contrast, a parameter that captures very little individual variability will not cause a substantial increase to model-fitting errors when fixed to the group mean, and will therefore have an RMSE value similar to the full four-parameter model.

**Figure 2:**
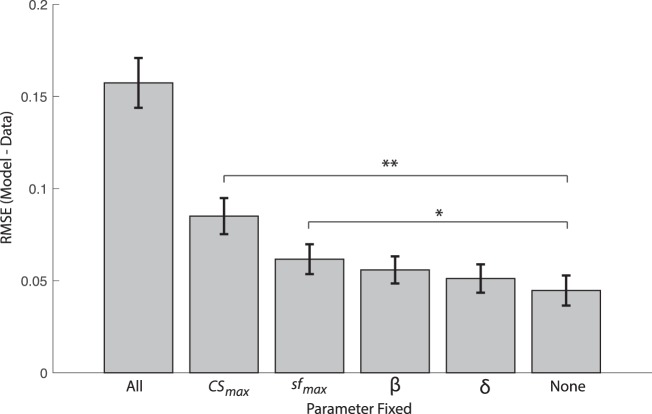
Bar plot showing the group mean of model-fitting errors, quantified as the root-mean-squared error, between logCS data and the best-fitting truncated log-parabola function in which select parameters were fixed to the group mean. “All” represents the case in which all parameters are fixed to group mean and none are free to vary in fitting individual-subject data (an upper bound on modeling errors). “None” represents the case of the full model with four free parameters (a lower bound on modeling errors). Each other case represents a partial model with three free parameters in which the designated parameter was fixed to the group mean. Error bars represent standard error of the mean. ^*^p < 0.05, ^**^p < 0.01.

Figure 2 shows group-averaged RMSE values across all three CSF test types for each partial model (in which a specific parameter is fixed) as well as the full model in which none of the parameters were fixed (i.e., all four parameters were free to vary) and an extreme case of the null model in which all of the parameters were fixed to the group mean (and were thus not free to vary for any individual subject). The first key observation is that model fitting suffers most when peak CS is fixed to the group mean (RMSE increases by about a factor of 2), because it prevents the model from shifting the CSF vertically along the logCS axis to account for individual differences in peak sensitivity. By this account, peak CS is clearly the most functionally relevant single parameter, followed by peak SF and more remotely by bandwidth and truncation. Comparing distributions of the RMSE of each partial model to the full model through *t*-tests reveals that only peak CS, *t*(246) = 5.38, *p* < 0.001, and peak SF, *t*(246) = 2.51, *p* = 0.012, had significantly worse RMSE values. Fixing bandwidth had a marginal impact, *t*(246) = 1.72, *p* = 0.09, and fixing truncation had a minimal impact on the RMSE of model fits, *t*(246) = 0.98, *p* = 0.33. The fact that model fitting is robust to cases in which select parameters are fixed to the group mean supports the idea that a template CSF may be adapted from group data to account for the global shape of the CSF and then subsequently adjusted according to just the two most relevant parameters (i.e., peak CS and peak SF) to potentially provide an accurate fit of individual data (Chung & Legge, 2016).

### Correlations between CSF parameters and auxiliary visual measures

**Figure 3:**
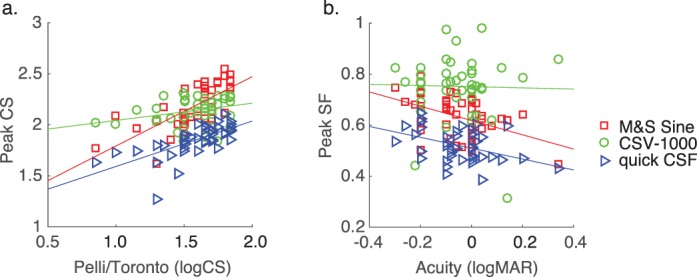
Scatterplots showing, for three different CSF tests, the relationships (a) between letter CS and fitted peak CS parameter and (b) between far acuity and fitted peak SF parameter. Lines represent least-squares linear model fits to the data.

Finally, a central issue of the study is to assess the relationship between CSF model parameters and auxiliary measures of visual function. Table 4 shows correlation coefficients relating high-contrast near acuity (40 cm), far acuity (3 m), and letter CS to each parameter of the best-fitting truncated log-parabola function, as well as summary statistics (AULCSF and high-SF cutoff). Far acuity generally had a stronger relationship to CSF parameters than near acuity, likely due to the fact that CSF tests were also done at a distance (≥8 ft). However, letter CS tended to have the highest correlations overall, particularly with the peak-CS parameter (CS_max_). We found a statistically significant correlation between letter CS and peak CS for two of the three tests, as shown in Figure 3. Far acuity had a significant negative correlation with peak SF and high-SF cutoff values for the Sine and qCSF tests but, again, not for the CSV-1000. While the CSV-1000 produced parameter estimates that were not significantly correlated with acuity and letter CS, the relationship between peak CS and far acuity trended in the expected direction.

The bandwidth parameter was less consistent than peak CS and peak SF, and showed no relationship to auxiliary measures for the Sine and CSV-1000 tests—but interestingly, it did show a relatively high correlation to far acuity and letter CS for the qCSF test. The truncation parameter was also a mixed bag, showing some significant correlations among tests and measures, but was generally less consistent than peak CS and peak SF. The strongest correlations were found for summary measures—AULCSF and high-SF cutoff—likely because these measures represent the composite influence of all four parameters and are therefore less noisy than any single measure in capturing visual function. Each summary measure showed effects similar to peak CS and peak SF, namely, a negative relationship with acuity and a positive relationship with letter CS, particularly for the Sine and qCSF tests. By comparison, the CSV-1000 test was less consistent, perhaps because it produces highly quantized values of logCS, owing to the fact that it represented only eight distinct levels of contrast for each SF. We return to this point in the Discussion to help explain potential limitations of the methodology used by the CSV-1000 in comparison to the other tests we examined.

## Predictive model

The results outlined so far can be distilled to three key insights. First, there are various approaches to estimating the CSF of an individual subject, and each type of test produces a distinct estimate of CSF parameters according to a test-specific template, as seen by group averages in Figure 1. These generalized test-specific differences in the shape of the CSF likely relate to methodological differences in task, threshold estimation procedure, and visual-target characteristics, among other factors (Woods & Wood, 1995). Second, some functional parameters of the truncated log-parabola model, such as peak CS and peak SF, appear to be more important than others for characterizing individual differences in CS. Our data show that the bandwidth and truncation parameters are relatively uninformative across subjects, consistent with the previous finding that bandwidth is effectively invariant between groups with normal and low vision (Chung & Legge, 2016). In other words, individual-subject data can be well captured even when these two parameters are no longer free to vary, leaving peak CS and peak SF as the only necessary model parameters to fit individual data. Lastly, peak CS and peak SF show a significant linear relationship with other independent empirical measures of visual function, including (respectively) letter CS and high-contrast visual acuity. Taken together, these findings offer a concrete recipe for predicting the full CSF on an individual basis with a deterministic predictive model that has essentially zero free parameters.

### Method and procedure

The procedure of the predictive model is to first establish a standard template CSF derived from normative group data that is distinct for a given test type (due to aforementioned mean differences among tests), and then to shift this template left, right, up, or down according to performance on other auxiliary measures of visual function. Here, the template CSF is defined by the truncated log-parabola model (see Equations 1 and 2), in which parameters for bandwidth and truncation are fixed via mean estimation from normative group data:

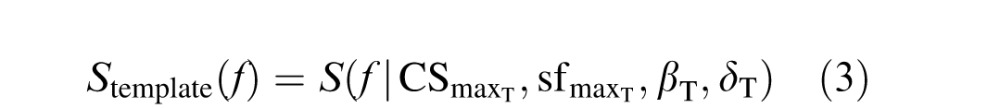


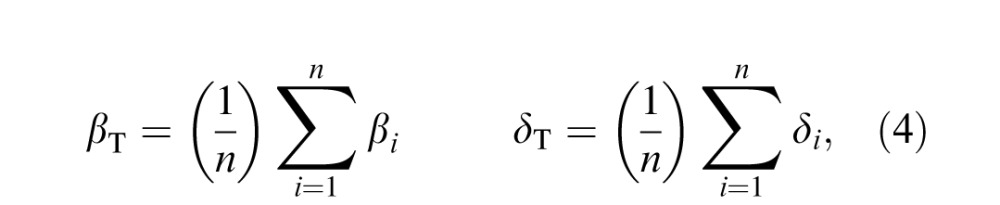
where *n* is the total number of subjects, subscript *i* represents the *i*th subject in the group, and subscript T indicates the parameter set defining the CSF template. Template parameters for peak CS and peak SF are not fixed to the group mean but instead vary according to independent measurements of letter CS and far visual acuity, respectively, following the linear models:

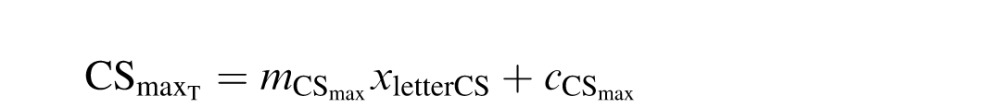


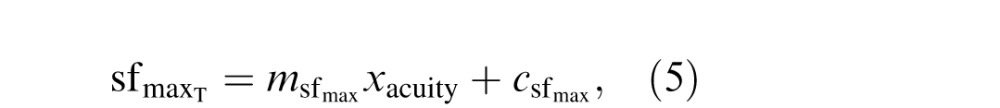
where *x*_letterCS_ and *x*_acuity_ represent empirical measurements of performance on the Pelli–Toronto letter CS and ETDRS high-contrast acuity tests, *m* is the slope of the best-fitting linear function, and *c* is the intercept term. Least-squares regression is used to estimate the slope and intercept terms from group data, and hence used to predict template parameters of peak CS and peak SF for any subject not contained within the normative group data set. As such, the entire predictive model is determined by just six parameters—*β*_T_, *δ*_T_, 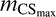 , 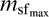 , 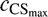 , and 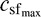 —which capture group-level patterns and relationships in the data. Thus, predicting individual data from this model is deterministic and involves effectively zero free parameters. More generally, once a CSF template and its six parameters are estimated from normative data, the input to the model to predict the full CSF curve of an individual is simply two empirically measured values: letter CS and high-contrast acuity.

To evaluate the performance of the predictive model on each individual subject, we use a leave-one-out procedure that estimates six template parameters from the group cohort that includes all subjects except the one to be predicted. The model is then evaluated for the left-out subject according to that subject's individual measurements of letter CS and far acuity to predict parameters of the subject's CSF. By evaluating the parameterized log-parabola function at the SFs specified by the original test (i.e., 1.5, 3.0, 6.0, 12.0, and 18.0 c/°), we derive predictions to be compared directly to the raw logCS measurements.

Model performance was evaluated according to RMSE between raw and predicted values (Equation 6), which was compared directly to test–retest errors (Equation 7) and errors in fitting the full CSF model with four free parameters (Equation 8):

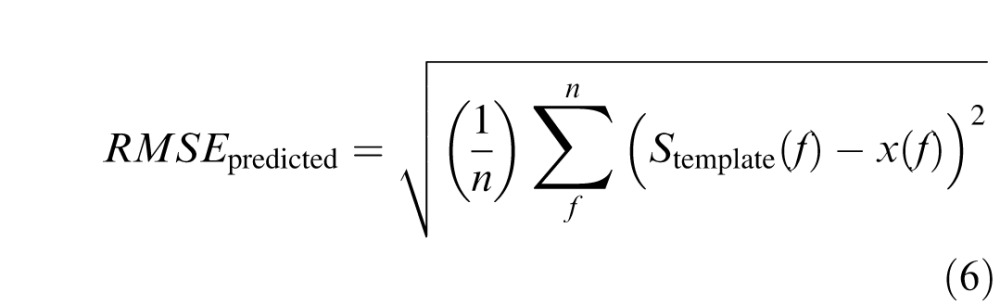


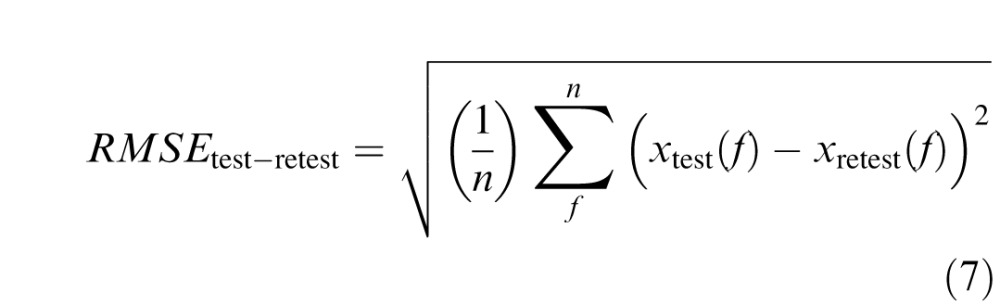


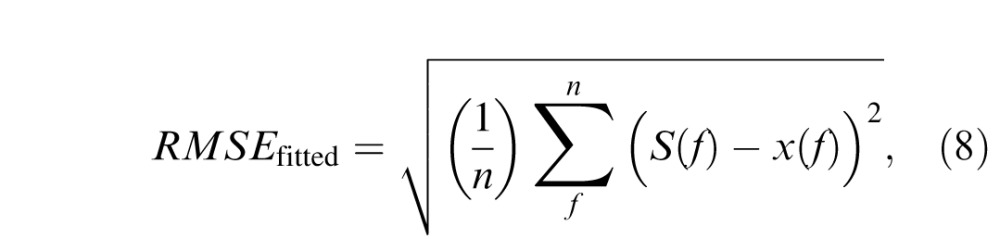
where *n* is the number of measured frequency-specific data points (i.e., 1.5, 3.0, 6.0, 12.0, and 18.0 c/°), *f* is an integer index for stepping through SF levels, *x*(*f*) represents actual measured data, *S*(*f*) represents frequency-specific values of the fitted CSF function (see Equations 1 and 2) and *S*_template_(*f*) represents frequency-specific values estimated from the predictive model (see Equations 3–5).

### Results

**Figure 4:**
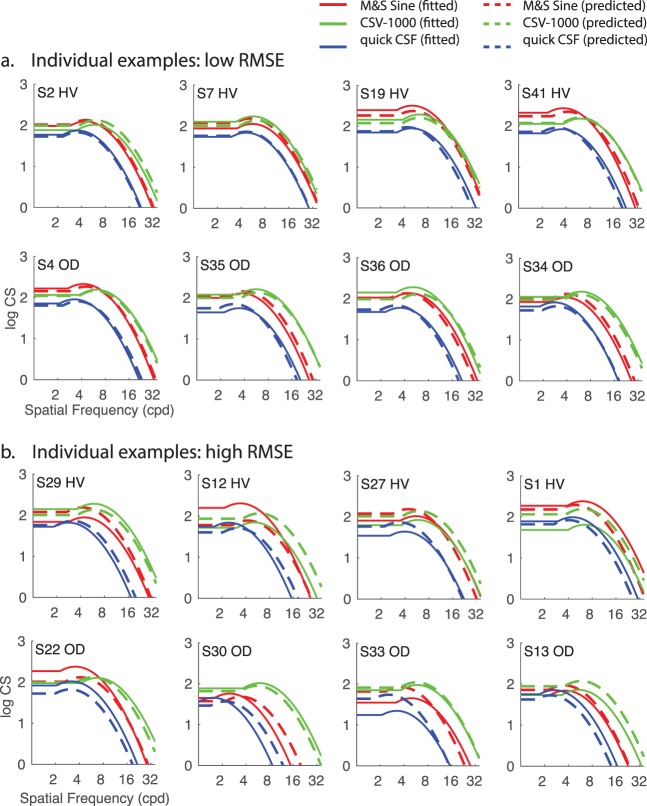
Individual plots of CSF curves either fitted with the full model with four free parameters (solid lines) or predicted with the zero-free-parameter model (dashed lines) for individuals (labeled S#) exemplary of (a) low error and (b) relatively high error. HV: healthy vision; OD: previous history of diagnosis of any ophthalmologic disease.

Not surprisingly, there was variability across the group in terms of how well the zero-free-parameter model could predict individual CSF curves. Figure 4 shows example subjects with relatively low error (Figure 4a) and relatively high error (Figure 4b). The source of this variability is not immediately clear, because the presence or absence of diagnosed ophthalmologic disease was not a predictive factor for RMSE_predicted_. For example, two-sample *t*-tests revealed no significant difference (all *p*s > 0.20 for each CSF test type) in RMSE_predicted_ between subjects with a prior history of ophthalmologic disease (OD; *n* = 14) and subjects (n = 29) without a prior history (HV; *n* = 29). There are several examples of low- and high-error predictions within each subgroup, as shown by organization in rows within Figure 4.

**Figure 5:**
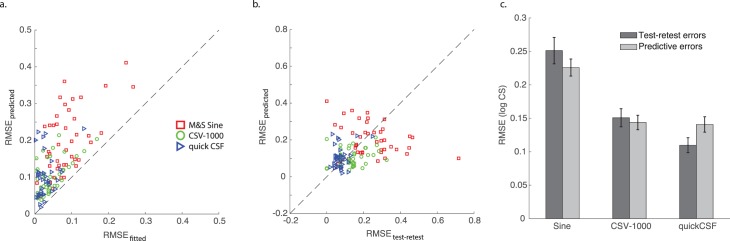
Scatterplot of RMSE_predicted_ (i.e., the zero-free-parameter model's predictions) on the y-axis with (a) RMSE_fitted_ (i.e., four free parameters) on the x-axis and (b) RMSE_test-retest_ on the x-axis, representing intrinsic test reliability. (c) The group mean RMSE_test-retest_ values are comparable to RMSE_predicted_ for each of three examined CSF tests. Error bars represent standard error of the mean.

As discussed previously, a key benchmark for interpreting the degree of error in model predictions across subjects is to compare them directly to both RMSE_test-retest_ (the consistency of the test on repeated measurements), which would determine whether they are within the same range of reliability as the instrument or test itself, and RMSE_fitted_ (with four free parameters to vary), which provides an effective upper bound on how well the truncated log-parabola functions can fit the data in the first place. The scatterplot in Figure 5a shows that all values of RMSE_predicted_ fall above the unity line with RMSE_fitted_, highlighting the fact that the ability to predict individual performance with this model is constrained inherently by the quality of fit for the full four-parameter CSF model. In other words, individuals with high RMSE_fitted_ will necessarily have greater RMSE_predicted_ values due to the fact that the zero-free-parameter model is couched in the same parametric form (i.e., the truncated log parabola), and this model is shown to fit the particular subject only so well in the first place. The variability in the ability of the full CSF model to fit individual subjects is clearly a driving factor in explaining variability in the performance of the predictive model.

By comparison, as shown in Figure 5b, the RMSE_predicted_ and RMSE_test-retest_ values are uncorrelated across individuals, and in fact have a similar underlying mean distribution. For example, for the Sine test the mean RMSE_test-retest_ across individuals was 0.25 (*SD* = 0.13) and the mean RMSE_predicted_ was 0.23 (*SD* = 0.08), and a *t*-test revealed no significant difference between these distributions, *t*(42) = 1.02, *p* = 0.32. Likewise, for the CSV-1000 the mean RMSE_test-retest_ across individuals was 0.15 (*SD* = 0.09) and the mean RMSE_predicted_ was 0.14 (*SD* = 0.07), and a *t*-test revealed no significant difference between these distributions, *t*(42) = 0.45, *p* = 0.65. For the qCSF test the mean RMSE_test-retest_ across individuals was 0.11 (*SD* = 0.07) and the mean RMSE_predicted_ was 0.14 (*SD* = 0.08), and a *t*-test revealed marginal statistical significance between the distributions, *t*(42) = −2.0, *p* = 0.051. Even accounting for the fact that error is introduced to model predictions directly via errors in parametric function fitting, as demonstrated in Figure 5a, this result demonstrates that predictive errors are nonetheless within the same range as measurement errors assessed on test and retest (Figure 5c).

It is important to underscore the point here that this level of prediction accuracy is achieved with a template CSF that is shifted according to independent measures of visual function, and is *not* achieved via optimized least-squares fitting of the parametric CSF model to individual-subject data. In theory, the CSF for any number of future subjects can be predicted with this same model by simply collecting measurements for letter CS and high-contrast acuity under similar testing conditions and plugging them into Equations 3–5. Of course, for future application a larger set of normative data would be desirable to produce even more precise estimates of test-specific CSF templates. For reference, the six-parameter template CSF values for each test type are reported in Table 5.

**Table 5:**
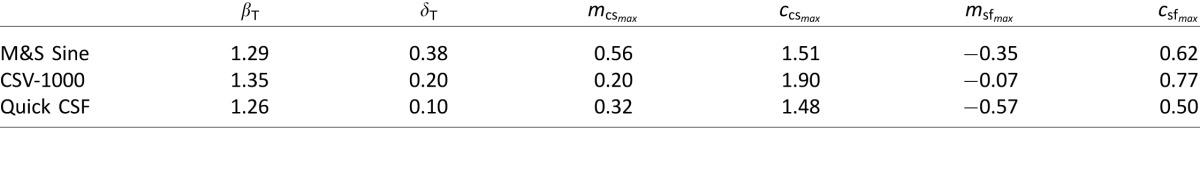
Template parameters fitted to normative (group) data, according to Equations 3–5.

## Discussion

This study was designed to evaluate several aspects of CS data within a heterogeneous cohort of subjects with minimal visual impairment, while focusing on three distinct tests designed to measure the CSF (M&S Sine test, CSV-1000, and quick CSF). Our analysis focused on several factors, including test–retest repeatability, parametric CSF model fitting and comparisons among test types, reliability of individual functional parameters for describing individual differences, and correlations among functional parameters with auxiliary measures of visual function, and culminated with the development of a predictive model built directly from collective findings in these analyses. This approach was motivated by very recent work by Chung and Legge (2016), who proposed the idea that results from any given CSF test may be described by shifting a generic parametric CSF template horizontally and vertically along the log-log axes to account for individual differences in just two dimensions—peak CS and peak SF.

In general, our choice of parametric curve to model the CSF (i.e., truncated log-parabola function) was suitable to account for the shape of the CSF derived from each test, and produced relatively small errors in curve fitting for most individuals, consistent with prior studies (Chung & Legge, 2016; Rohaly & Owsley, 1993). This function contains four free parameters that represent interpretable aspects of the CSF: peak SF and CS of the parabolic curve on the x- and y-axes, respectively, as well as the width of the curve and the canonical asymmetric plateau at lower frequencies (Lesmes et al., 2010; Watson & Ahumada, 2005). Interestingly, we found that fixing bandwidth and truncation parameters to the group mean, forbidding them to vary freely for individual subjects, did little to impair the overall performance of curve fitting (Figure 2). On the surface, this result supports the idea that an individual's CSF can be mostly approximated by shifts of a generic CSF template along the logCS and log-SF axes (Chung & Legge, 2016; Pelli et al., 1986).

The positive relationship between peak CS and letter CS is not a new discovery (Pelli et al., 1986), and in fact was likely important for early adoption of the Pelli–Robson chart for clinical testing of CS (Pelli et al., 1988). The negative relationship between peak SF and high-contrast acuity measurements represented in logMAR is a relatively more recent discovery (Chung & Legge, 2016), which we anticipated replicating in the present study. Indeed, we replicated both of these key findings for two out of the three CSF tests, with the exception of the CSV-1000 test (although it trended in the expected direction). This may be due to the fact that contrast levels in this test are highly quantized (only eight total levels), due to space limitations on the printed board and the importance of time efficiency for an examination designed for the clinic. This degree of quantization also likely played a role in the good test–retest repeatability for the CSV-1000, but on the other hand likely made it difficult to characterize subtle differences in CS across subjects.

Given these statistical relationships among functional parameters and auxiliary visual measures, we developed a generic modeling framework based on a template CSF defined by the truncated log-parabola function. The template CSF for a given test type is defined by just six parameters determined from normative group data, for instance, where bandwidth and truncation parameters are constants estimated from the group mean and where peak CS and peak SF vary for each individual by a linear function according to empirical estimates of letter CS and acuity. We see the principal innovation of this work as the formalization of a general-purpose parametric model that can predict an observer's individual CSF curve on the basis of standard and more basic clinical measurements with a suitable degree of accuracy. We supposed that a predictive model that could achieve accuracy within the range of test–retest reliability could serve two important purposes: (a) As a practical matter, such a model could be used to predict CSF curves for any arbitrary CSF test in which normative data were available, potentially allowing better comparisons among data sets in the literature and in the clinic; and (b) as a theoretical matter, it would suggest that there is such redundancy between CSF measurements and other common clinical measures that it would obviate the need to spend time estimating the full CSF curve in many clinical situations.

On this matter, our results affirm the notion that CSF tests are highly redundant and differ from each other primarily on the surface (e.g., in terms of large-scale differences in global shape or template) but deliver essentially the same information as other tests that are easier to use and more readily available, such as high-contrast ETDRS acuity and letter CS estimated from single unfiltered optotypes. Yet CSF tests do differ from each other in practically relevant ways, such as test–retest repeatability and the precision of threshold estimates. That these results were found in such a diverse cohort of individuals suggests that these findings should extend to most people within the range of relatively normal visual function. The degree to which this modeling framework would apply well to particular patient populations with more severe visual impairment is an important topic for future study. However, the study by Chung and Legge (2016) did use a low-vision population with much greater disability and found comparable results in terms of reporting that the bandwidth parameter was invariant between groups with low and normal vision and finding that peak SF was significantly correlated with high-contrast acuity. In their work, a parametric CSF template derived from observers with healthy vision was similar to that measured for those with low vision, suggesting invariance of the shape of the CSF for both groups of subjects. We presume that the results of the current method would extend favorably to this population as well, but this hypothesis remains to be tested.

While the results in the present article do suggest that in many cases, estimation of the full CSF function may produce redundant information with the more quickly and easily measured assessments of acuity and letter CS, it would be inappropriate to assert that less will always be more. As can be seen in Figure 1, there may be some information contained in the bandwidth and truncation parameters, as they differ substantially according to test type; however, a difficulty is that the information potentially provided from these measures may not rise beyond their reliability. Thus, for CSF tests to provide information beyond acuity and letter CS that is useful, they should be able to estimate these additional parameters with precision and reliability and to additionally show that these parameters are diagnostic of patient conditions and predictive of outcomes. On this count, we believe the quick CSF has proven the best potential in terms of delivering highly repeatable measurements and functional parameter estimates with less apparent noise as demonstrated by their uniquely strong relationship to auxiliary vision measures (see Table 4). Future research efforts will benefit by reproducing these results with respect to other CSF tests, and by assessing whether or not the predictive quality of our zero-free-parameter model is affected by the study of other patient groups with vision impairment or other serious ophthalmologic conditions.

For instance, one potential limitation of the present approach is the inability to characterize subtle changes to the shape of the CSF, in particular midfrequency notches that may result from retinal or optic-nerve disease, multiple sclerosis, or even optical abnormalities such as astigmatism (Apkarian, Tijssen, Spekreijse, & Regan, 1987; Regan et al., 1977; Regan, Bartol, Murray, & Beverley, 1982). This is due to the fact that the truncated log-parabola function, and in fact most parameterized CSFs (Watson & Ahumada, 2005), embodies a limited number of parameters to characterize key features of the CSF, such as the peak of the curve, its bandwidth, and the common asymmetry found at low frequencies. Lacking additional parameters to account for possible midfrequency notches, these functions would all effectively smooth over such deviations from the template CSF. As a practical matter, there is a potential cost to overfitting individual data, as fitting functions are designed to become more elaborate and highly parameterized to account for idiosyncratic deviations from the generalized template. While the set of commonly used functions that have four or five free parameters appear to strike a good balance between generalizability and parsimony, they may collectively suffer from insensitivity to potentially functionally relevant deviations from the assumed functional form. Hence, while we conclude that in many cases the CSF of an individual can be accurately measured (Lesmes et al., 2010) or even predicted, as shown in the present article, on the assumption of a specific underlying functional form of the CSF, these methods are not without potential limitations that should be regarded before use in clinical applications that aim to measure particular frequency-specific deficits in CS.

## Supplementary Material



Supplement 1
