# Supplementary figures and images for: Predicting individual contrast sensitivity functions from acuity and letter contrast sensitivity measurements
Source: J Vis. 2016 Dec 22;16(15):15. doi: 10.1167/16.15.15 (PMC5221673; doi:10.1167/16.15.15)

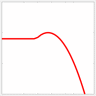

Supplement: Supplementary file 1 [file JOV-04993-2015-s02-ICON.gif]

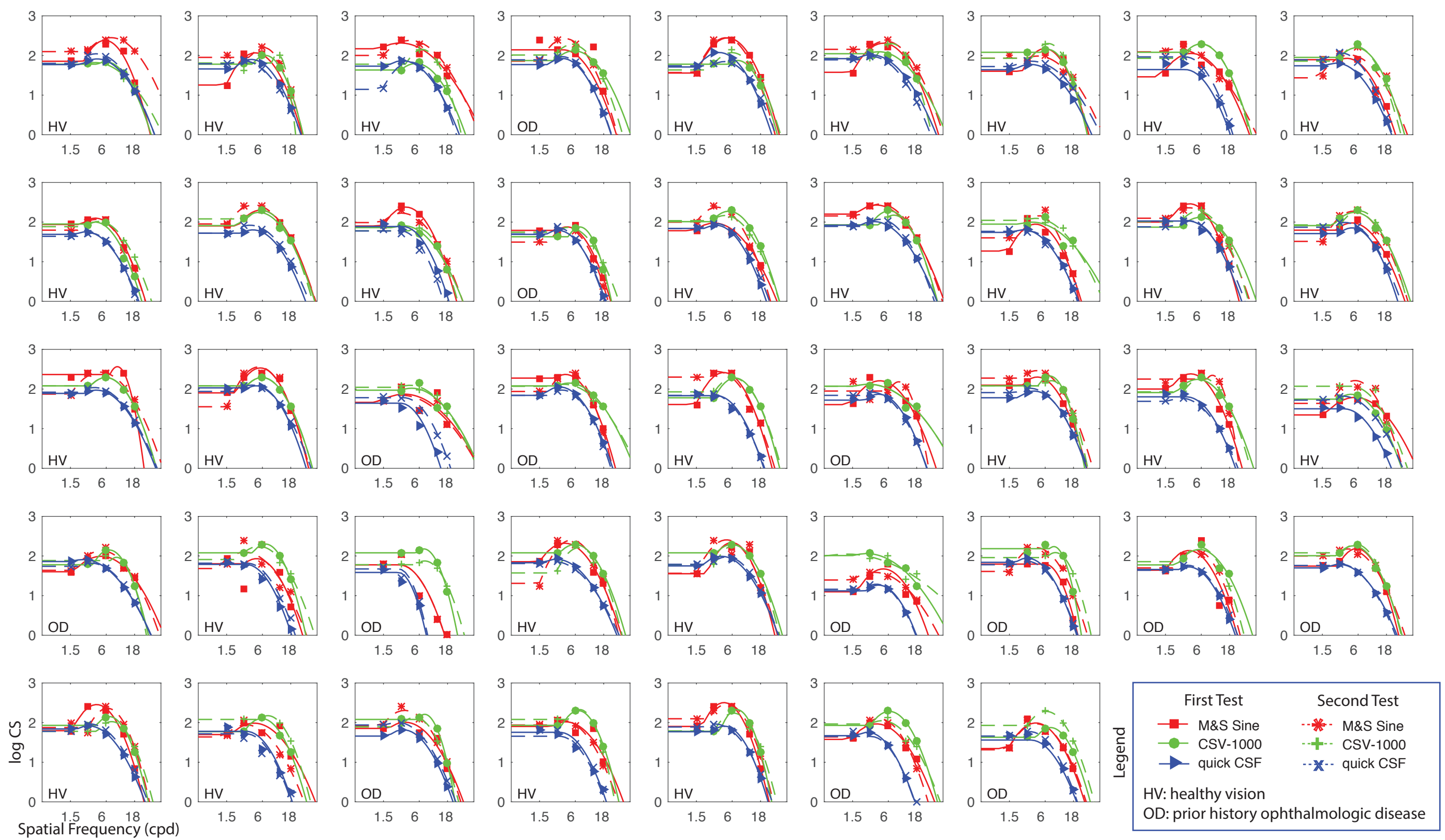

Supplement: Supplement 1 [file JOV-04993-2015-s01.pdf]
